# The Sample Copy Man

**Published:** 1878-02-20

**Authors:** 


### THE SAMPLE COPY MAN.

We have to add our testimony to the complaints that have come up from many of our exchanges, that we have been greatly imposed upon by the "sample copy man." For a one cent postal card he procures a journal worth 25 cents, besides the postage, which is two cents on every copy so mailed. We dislike to refuse these calls, but really it is a little too much to expect of us to submit longer to this tax. Our experience has shown that only about one per cent of all the number who have called for sample copies are ever heard from again.

